# Ecopharmacovigilance and pharmacovigilance: an analysis of environment-related reporting in VigiBase

**DOI:** 10.1007/s11356-026-37744-6

**Published:** 2026-04-15

**Authors:** Joseph Mitchell, José Maza Larrea, Ernest Dela Dzidzornu, Jayesh Pandit, Jerin Jose Cherian, Robert Massouh

**Affiliations:** 1 Department of Global Public Health, Karolinska Institutet, Stockholm, Sweden; 2 The ISoP Special Interest Group On Ecopharmacovigilance, London, UK; 3 Uppsala Monitoring Centre, Uppsala, Sweden; 4 Independent Researcher, Accra, Ghana; 5 Pharmacovigilance & Patient Safety Consultant, Nairobi, Kenya; 6 Indian Council of Medical Research, New Delhi, India; 7 GSK, London, UK

**Keywords:** Pharmacovigilance, Ecopharmacovigilance, Ecotoxicology, Ecopharmacology, Adverse events, Environmental exposure, MedDRA®

## Abstract

There is growing interest in the effects of pharmaceuticals in the environment. However, the focus when assessing the risk of pharmaceuticals in the environment is done in the pre-authorisation phase, and there is no standard approach to identify, report and collect suspected adverse events related to environmental exposure and impact. This study aims to describe the reporting of environment-related terms to VigiBase, the WHO global database of adverse event reports. A search was performed on VigiBase on all data up to July 1st, 2024, since the first report in 1967. The search was for reports with any medicine or vaccine in combination with a set of Preferred Terms identified as being environment-related by the authors. A descriptive analysis of reports with an environment-related Preferred Term was then performed. A total of 713 reports (from a total of 38,141,125) were identified with environment-related Preferred Terms, with a steady increase in reporting since the first report in 2001. Reports were received for a variety of medicinal groups and commonly alongside Preferred Terms potentially describing symptoms of those exposed. There was also a large percentage of reports from non-healthcare professionals. The number of environment-related reports in VigiBase is small but increasing. This overview highlights some important features of environment-related reports that require consideration in any future work. It would be beneficial to develop a framework to guide the reporting and analysis of environment-related adverse effects of medicines, although the eventual relationship to traditional pharmacovigilance is unclear.

**Supplementary Information:**

The online version contains supplementary material available at 10.1007/s11356-026-37744-6.

## Introduction

There is growing interest in the effects of pharmaceuticals in the environment and whilst ecopharmacovigilance is not a new concept, there is an increase in interest regarding the topic (Lunghi et al. 2024). This has occurred alongside the wider recognition of the importance of protecting the planet through environmentally friendly practices, including within healthcare (Bouzas-Monroy et al., 2022; Setoguchi et al. 2022; Velo and Moretti 2010). There has not been universal adoption of a definition of ecopharmacovigilance. However, ecopharmacovigilance has previously been defined as “the science and activities associated with the detection, evaluation, understanding and prevention of adverse effects of pharmaceuticals in the environment” (Holm et al., 2013). This can be interpreted as adverse effects on the environment itself, but also experienced by the humans and animals that come into contact with the pharmaceutically impacted environment (Velo and Moretti 2010). This definition highlights that although closely related and probably overlapping, the science of ecopharmacovigilance can be considered to be distinct from that of pharmacovigilance.

Pharmacovigilance practices ensure that medicinal products undergo continuous monitoring of their safety throughout their lifecycle, beginning with preclinical studies and extending to post-marketing surveillance (Potts et al. 2020; Lucas et al. 2022), with large databases collating reports and forming the backbone of post-marketing surveillance. There have been advancements in considering the impact of pharmaceuticals in the environment. Environmental Risk Assessments are predictive assessments of risks conducted for medicinal products (Holm et al., 2013), but these typically focus on the pre-authorisation period, thus usually conducted for new medications, with variability in how they are conducted (Ågerstrand et al. 2015; Jose et al. 2020; Gildemeister et al. 2023). In the post-authorisation phase however, there are limited activities evaluating the effects of chronic exposure in the environment (Vestel et al. 2016; Khan et al. 2020). There has also been progress in the capabilities of monitoring the presence of active pharmaceutical ingredients within the environment (Wilkinson et al. 2022), but there remains a scarcity of data available to perform risk assessments (Spilsbury et al. 2024) and further there is an imbalance of monitoring between countries (Khan et al. 2020; Wilkinson et al. 2022).

VigiBase, the WHO global database of adverse event reports for medicines and vaccines, receives individual case reports relating to a human subject from members of the WHO Programme of International Drug Monitoring. VigiBase contains over 38 million reports of suspected adverse events and the WHO Programme of International Drug Monitoring network covers over 99% of the global population (Uppsala Monitoring Centre, n.d.). For ecopharmacovigilance and pharmaceuticals in the environment there is no standardised approach to identify and report adverse events whether the endpoint is the environment or a human or animal that has been in contact with a pharmaceutically impacted environment. This reduces the possibility to identify and act upon potential adverse events related to environmental exposure (Holm et al., 2013). Unlike traditional pharmacovigilance, there is currently no specialised database to collect reports of suspected adverse events related to pharmaceuticals in the environment, with results often disparately spread amongst published scientific literature (Holm et al., 2013).

Pharmaceuticals are typically found at low concentrations (aus der Beek et al. 2016; Wilkinson et al. 2022) and most pharmaceuticals may pose a relatively low environmental risk (Gunnarsson et al. 2019). However, there are examples where the adverse effects of medicines on the environment have been observed, and action taken to reduce this impact. Adverse events have been observed in animals (Bouzas-Monroy et al., 2022; Khan et al. 2020; Velo and Moretti 2010). The decline of vulture populations exposed to diclofenac (Oaks et al. 2004; Taggart et al. 2007; Nethathe et al. 2021) from feeding on dead cattle treated with the drug is a noteworthy example of the potential impact a drug, given sufficient exposure, can have on an unintentionally exposed species. Another relevant example are fish exposed to environmental oestrogens (Kidd et al. 2007) where effects may be observed at very low concentrations of the pharmaceutical. Further, the role of environmental exposure to antibiotics; released from patients, animals and manufacturing plants; in the development of antibiotic resistance in both animals and humans is widely discussed and researched(Bengtsson-Palme et al. 2018; Booton et al. 2021; Mitchell et al. 2021).There is also recent acknowledgement of the wider effects of pharmaceuticals on the environment with one example of this being the restrictions regarding the use of desflurane due to its global warming potential (Bracco and Bozzer 2024).

A complicating factor with regard to using traditional pharmacovigilance to investigate unexpected exposures to pharmaceuticals in the environment, and any adverse events, is that these exposures are often to a variety of products rather than one or a few products, as is more typical in traditional pharmacovigilance (Holm et al., 2013; Velo and Moretti 2010). Furthermore, the traditional post-marketing surveillance of medicinal products relies on both healthcare professionals and those experiencing the adverse events, their interactions and subsequent spontaneous reporting. The level of suspicion of environmental exposure and subsequent interactions between healthcare professionals and those exposed are often missing in ecopharmacovigilance (Holm et al., 2013). This suggests that traditional pharmacovigilance alone may not be suited to detect the impact of pharmaceuticals in the environment. More targeted and region-specific approaches to measure environmental concentrations may be required to support a lifecycle approach to detection and analysis of the impact of pharmaceuticals in the environment (Wang et al., 2018, 2019). For example, the European Union-wide monitoring programmes for chemical substances, which includes pharmaceuticals, under the Water Framework Directive is a potential source of such data for the limited number of pharmaceuticals within the scope of the WATCH list (Colzani et al. 2024).

There are limitations of post-marketing pharmacovigilance in supporting the analysis of the effects of pharmaceuticals in the environment and difficulties establishing exposure of humans to pharmaceuticals in the environment (Holm et al., 2013). However, spontaneous reporting systems are a key aspect of traditional pharmacovigilance (Sartori et al. 2023). There have been several reviews of the need and challenges for pharmacovigilance as well as more local implementation of ecopharmacovigilance practices (Holm et al., 2013; Velo and Moretti 2010; Wang et al., 2017, 2019). But there has been no assessment as to whether environment-related reports are received by large pharmacovigilance database and how they are reported. This is crucial to understand current reporting practices and to help determine whether they could be source to complement a lifecycle approach of adverse events in relation to ecopharmacovigilance. Therefore, this study aims to describe and analyse the reporting of environment-related terms in VigiBase. Furthermore, it aims to analyse these reports and discuss implications for future research using pharmacovigilance data in the field of ecopharmacovigilance.

## Methods

### VigiBase search

A search of VigiBase was performed on a frozen dataset up to July 1 st, 2024, that included reports dating back to 1967. The database is designed to collect reports for humans, with an identifiable human subject being a requirement for a valid case report. Further basic requirements for a valid report are an identifiable reporter, a suspected medicinal product and an adverse event. Therefore, it is most likely that the case reports in VigiBase identified by this search describe an patient who has reported an environment-related adverse event with an exposure to a medicinal product. The information in VigiBase comes from a variety of sources, and the probability that the suspected adverse effect is drug-related is not the same in all cases. The dataset included both reports for medicines and vaccines and was automatically deduplicated by vigiMatch (Tregunno et al. 2014). The search was for the reported adverse events, and it was based on the Preferred Terms (PTs) within the MedDRA® Higher Level Terms that were considered environment related. MedDRA® Higher Level Terms are based upon factors such as aetiology (as is the case of environment-related PTs), anatomy, physiology, pathology and function(MedDRA, n.d.). MedDRA®, the Medical Dictionary for Regulatory Activities terminology, is the international medical terminology developed under the auspices of the International Council for Harmonisation of Technical Requirements for Pharmaceuticals for Human Use. The Higher Level Terms used were, “Non-occupational environmental exposures”, “Occupational exposures”, “Non-occupational and unspecified environmental problems” and “Occupational environmental exposure”. The authors also searched the MedDRA® medical dictionary, version 27.0, to find other PTs that may be of relevance to the topic area. Two authors identified the relevant PTs, which were then reviewed and adjudicated by all other authors. If the authors agreed that a term was not potentially related to environmental exposure, then the PT was removed from the search. Most PTs included in the search were related to route or cause of exposure. The exception to this is the PT Idiopathic environmental intolerance, this PT refers to a condition where exposure to one or more environmental substances leads to wide ranging symptoms. (Binkley 2023)The full list of PTs reviewed by the co-authors is shown in Table 1.

**Table 1:** MedDRA® Preferred Terms that were identified and reviewed for inclusion as an environment-related Preferred Term to use in the search in VigiBase

	Environment-related Preferred Term	Exclude
Non-occupational environmental exposures (Higher Level Term)		
Environmental exposure	X	
Exposure to allergen		X
Exposure to chemical pollution	X	
Exposure to contaminated air	X	
Exposure to contaminated water	X	
Exposure to extreme temperature		X
Exposure to noise		X
Exposure to polluted soil	X	
Heavy exposure to ultraviolet light		X
Occupational exposures (Higher Level Term)		
Occupational exposure to air contaminants		X
Occupational exposure to communicable disease		X
Occupational exposure to dust		X
Occupational exposure to extreme temperature		X
Occupational exposure to noise		X
Occupational exposure to product		X
Occupational exposure to radiation		X
Occupational exposure to SARS-CoV-2		X
Occupational exposure to sunlight		X
Occupational exposure to toxic agent		X
Occupational exposure to vibration		X
Non-occupational and unspecified environmental problems (Higher Level Term)		
Flooding	X	
Food contamination	X	
Pollution	X	
Poor sanitation	X	
Water pollution	X	
Occupational environmental problems (Higher Level Term)		
Occupational physical problem		X
Occupational problem environmental		X
Other Preferred Terms (Higher Level Term)		
Exposure to toxic agent		X
Idiopathic environmental intolerance	X	
Manufacturing materials contamination		X
Product contamination		X
Product contamination chemical		X
Product contamination physical		X
Wound contamination		X
Exposure to unspecified agent		X
Suspected product contamination		X

### Patient and public involvement

There was no patient or public involvement in the research.

### Data extraction

The following variables were available to be extracted from the reports; age group of the subject of the report; sex of the subject of the report; suspected or interacting medicinal products, as defined by the reporter or those sending the report; concomitant medicinal products; the Anatomical Therapeutic Chemical (ATC) Classification of the medicinal products (WHO, n.d.); MedDRA® PTs; the qualification of the reporter; if the report was marked by the reporter or those sending the report as being serious; the year of reporting; and the country from which the report originated which were then grouped together based on the WHO regions.

The ATC of the medicinal products was determined to the first (e.g., J – Antiinfectives for systemic use) and second level (e.g., J01 – Antibacterials for systemic use). There can be more than one ATC per medicinal product. The seriousness of a report is identified by the reporter and their judgement as to whether or not it meets one of the seriousness criteria defined by the International Conference of Harmonisation. These criteria are, “Death”, “Life threatening”, “Caused or prolonged hospitalisation”, “Persistent or significant disability”, “Congenital birth defect” or is “Medically important condition for another reason”(International Conference on Harmonisation of Technical Requirements for Registration of Pharmaceuticals for Human Use., 2003). Both the seriousness criteria and qualification of the reporter can have more than one response for each report.

### Analysis

Analysis was performed using Microsoft Excel and RStudio and a descriptive analysis of the search results of environment-related PTs was performed. The percentage of each seriousness criteria was calculated on the number of reports marked as serious and reports with an unknown reporter qualification were excluded from the percentage calculations for each reporter type. Reporting for the ATC codes was analysed as a percentage of the total ATC codes reported rather than the total number of reports. The percentage of seriousness criteria was calculated based on the total number of reports that were marked as serious, rather than all cases.

## Results

In total, the frozen VigiBase dataset contained 38,141,125 reports, and 713 reports included an environment-related PT included in the search term. Table 2 shows the case characteristics of reports identified by the search. Most of the reports were identified as being serious, with a total of 524 (73.5%) marked as serious. There were also high levels of reporting from non-healthcare professionals, with the most common reporter qualification being “Consumers or non-healthcare professionals” (n = 328, 55.1%), followed by “Lawyer” (n = 124, 17.4%). Most reports were from countries within two of the six WHO regions, namely the Region of the Americas (82.2%) and the European Region (17.0%).

**Table 2:** Characteristics of reports with environment-related Preferred Terms in VigiBase as of July 1 st, 2024

		Environmental exposure (n = 713)
Age Group (%)	0—27 days	0 (0.0)
	28 days – 23 months	9 (1.9)
	2–11 years	9 (1.9)
	12—17 years	5 (1.1)
	18–44 years	156 (33.3)
	45–64 years	155 (33.1)
	65—74 years	72 (15.4)
	75 years or more	62 (13.2)
	*Unknown*	*245 (34.4)*
Sex (%)	Male	257 (39.5)
	Female	394 (60.5)
	*Unknown*	*62 (8.7)*
Serious (%)		524 (73.5)
Seriousness criteria* (%)	Death	115 (21.9)
	Life threatening	28 (5.3)
	Caused/prolonged hospitalisation	141 (26.9)
	Disabling/incapacitating	141 (26.9)
	Other medically important condition	361 (68.9)
Country of report by WHO region	African Region	0 (0.0)
	Eastern Mediterranean Region	1 (0.1)
	European Region	121 (17.0)
	Region of the Americas	586 (82.2)
	South-East Asia Region	0 (0.0)
	Western Pacific Region	5 (0.7)
Reporter Qualification* (%)	Physician	82 (13.8)
	Pharmacist	26 (4.4)
	Other healthcare professional	69 (11.6)
	Consumer or non-healthcare professional	328 (55.1)
	Lawyer	124 (17.4)
	*Unknown*	*108 (15.1)*

**Fig. 1:**
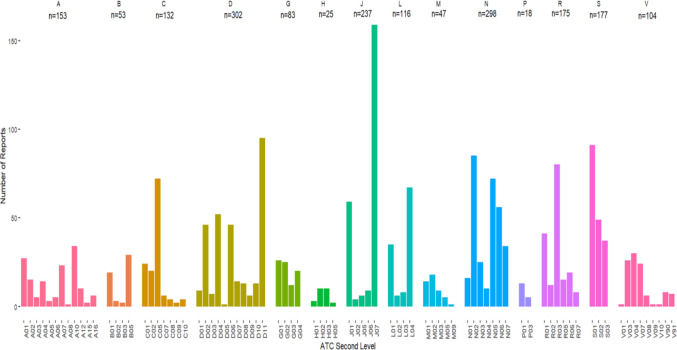
Number of medicinal products, grouped to the second ATC level in VigiBase as of July 1 st, 2024, reports with an environment-related Preferred Term

Of the 713 reports with an environment-related PT, there were 329 different medicinal products reported a total of 1,003 times with 1,968 ATC codes. The median number of medicinal products as suspected or interacting product, per report was 1 (Q1- 1, Q3–1). The reported medications were from a wide variety of the ATC code classifications (Fig. 1 and Supplementary Table 1). The most common ATC (second level) to receive reports was J07 – Vaccines (n = 159), followed by D11 – Other dermatological preparations (n = 95), S01—Ophthalmologicals (n = 91), N02 – Analgesics (n = 85) and R03 – Drugs for Obstructive airway diseases (n = 80). The highest number of reports by ATC first level was seen for ATC group D – Dermatologicals (n = 302), N – Nervous system (n = 298), J – Antiinfectives for systemic use (n = 237), S – Sensory Organs (n = 177) and R – Respiratory system (n = 175). There were also concomitant medications included in 204 of the reports, with 343 different concomitants reported a total of 974 times. The most common concomitant medications were salbutamol (n = 23), paracetamol (n = 20), atorvastatin and levothyroxine (both n = 18) and omeprazole and tiotropium (both n = 16).

**Fig. 2:**
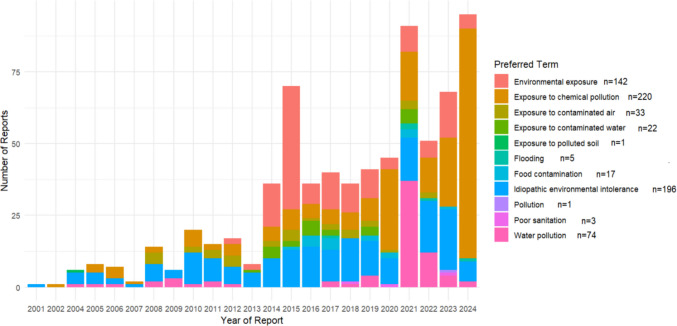
Environmental-related Preferred Terms reported to VigiBase, by year, as of July 1.^st^, 2024

A total of 714 Preferred Terms in environmental exposure PTs were reported within the 713 reports. The PT with the most reports was “Exposure to chemical pollution” (n = 220), followed by “Idiopathic environmental intolerance” (n = 196), “Environmental exposure” (n = 142), “Water pollution” (n = 74), “Exposure to contaminated air” (n = 33), “Exposure to contaminated water” (n = 22), “Food contamination” (n = 17), “Flooding” (n = 5), “Poor sanitation” (n = 3) and “Exposure to polluted soil” and “Pollution” (both n = 1). There has been a steady increase in reporting of these terms since the first report in 2001 until the time of data extraction (Fig. 2). There does appear to be a variation in the most commonly reported terms each year as well, with some years, in particular, seeing an increase in reports for a specific PT, such as, “Environmental exposure” in 2015, “Water pollution” in 2021 and “Exposure to chemical pollution” in 2024. These numbers are small and are thus open to small changes leading a relatively large variation but some PTs such as “Exposure to pollution” and “Idiopathic environmental intolerance” do seem to have a more consistent annual increase in reports.

**Fig. 3:**
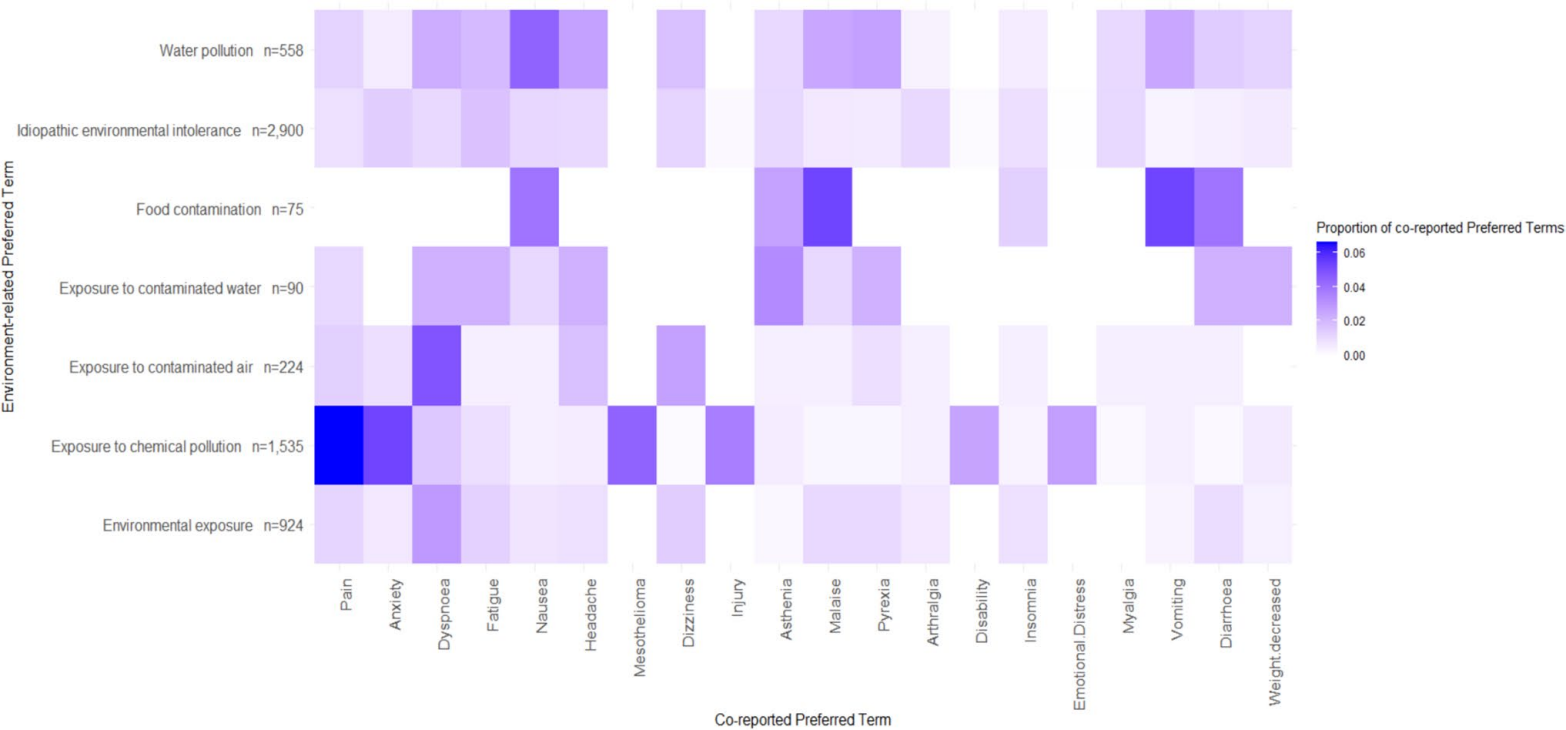
Heatmap showing the proportion of 20 most co-reported preferred terms overall, for each of the environment-related preferred terms in VigiBase reports as of July 1 st, 2024. N.B. The counts next to the environment-related Preferred Terms is the total number of co-reported terms for each of the Preferred Terms. The Preferred Terms "Exposure to polluted soil", "Flooding", "Pollution" and "Poor sanitation" were excluded from this figure as there were too few co-reported terms

A total of 1,665 PTs were co-reported 6,320 times. The most commonly co-reported Preferred Terms seemed to be related to patient symptomatology, with the top 10 co-reported terms being “Pain” (n = 149), “Anxiety” (n = 134), “Dyspnoea” (n = 107), “Fatigue” (n = 90), “Nausea” (n = 78), “Headache” and “Mesothelioma” (both, n = 69), “Dizziness” (n = 67), “Injury” (n = 62) and “Asthenia” (n = 55). Figure 3 shows how the 20 most co-reported PTs overall were reported with the environment-related PTs as a proportion of the total co-reported PTs. The PTs "Exposure to polluted soil", "Flooding", "Pollution" and "Poor sanitation" were excluded from this figure as they had too few total counts of co-reported PTs (n = 4, 12, 10 and 13, respectively). “Pain” in combination with “Exposure to chemical pollution”, was co-reported with the highest proportion of reports based on the environment-related PT. It is also noticeable that in environment-related PTs with a stated form of exposure the most co-reported PTs were typically consistent with that type of exposure. For example, “Food contamination” has a high proportion of reporting with “Nausea”, “Malaise”, “Vomiting” and “Diarrhoea” and “Exposure to contaminated air is most co-reported with “Dyspnoea”.

## Discussion

This study, for the first time, describes environment-related reporting of adverse events in VigiBase, an international pharmacovigilance database. The study provides an overview of the adverse events reported and the characteristics of these reports. Overall, the level of reporting remains small, but is increasing since the first report in 2001, with reports from a wide range of medicinal products, based on the ATC classification, and most commonly reported alongside PTs related to symptomatology.

With only 713 cases identified, environment-related terms constitute a very small number of the total reports in VigiBase (~ 0.00002%). However, this is not unexpected, given that this area falls outside the core area of pharmacovigilance and interest in the topic is relatively new. This is further compounded by the identified difficulties in stimulating reporting. This includes reliably identifying the exposure, attributing the exposure to a certain pharmaceutical product or products and a lack of suspicion from those who are exposed and to those to whom they may present to with their symptoms (Holm et al., 2013). Furthermore, to support a true lifecycle approach to ecopharmacovigilance, robust local testing of environmental concentrations is required to analyse levels of pharmaceuticals in the environment, which falls outside of the scope of pharmacovigilance. However, having a standardised approach to reporting suspected adverse events and collating them in one location, whether this is VigiBase or not, could be beneficial in identifying environment-related adverse events (Holm et al., 2013). A clear framework of reporting of adverse events related to pharmaceuticals in the environment may help to standardise and stimulate reporting and analysis of suspected adverse events. For example, taking steps such as the adoption of ecopharmacovigilance into the MedDRA® dictionary, either within the System Organ Class framework or as a Standardised MedDRA® Query will help to standardise and bring it in line with traditional pharmacovigilance. Such a framework would require the input of the multitude of stakeholders in ecopharmacovigilance, which includes but is not limited to, politicians, regulators, the pharmaceutical industry, scientists, healthcare professionals and the wider public (Holm et al., 2013; Lunghi et al. 2024). A future approach to identify potential adverse effects could leverage the strengths of the pharmacovigilance system as a complement to specific monitoring of environmental factors. With such an effort potentially requiring and benefitting from further collaborative efforts between health and environment agencies as well as other stakeholders. Such efforts would also be in line with calls to consider pharmacovigilance as a ‘one health’ topic that considers the interconnected effects of pharmaceuticals on humans, animals and the environment (Dikshit 2024; García et al. 2024).

It is also important to reflect upon the identified reporting patterns, with a general increase over time, although we were not able to put this into context with the overall growth of VigiBase over time. Broadly, the environment-related PTs were co-reported with other PTs that could reasonably expected, for example, “Exposure to contaminated air had a relatively high proportion of “Dyspnoea” as a co-reported term. Most reports were from non-healthcare professionals, with most coming from consumers or non-healthcare professionals (55.1%) and with a relatively large contribution from lawyers (17.4%), which is high compared to previous non-environment based studies of VigiBase data (Salem et al. 2018; Watson et al. 2022; Zhang et al. 2024). It could be speculated that this may highlight a medico-legal aspect, with parallels to other environmental exposures, but it is more important to consider the potential implications for why and how reports can be submitted (Härmark et al. 2015; Gauffin et al. 2023). Another unexplained, but potentially important, aspect is the high level of serious reports, with the most common reason for seriousness being “Other medically important condition” (68.9%). Future work to try and unravel the contents and relevance of these reports, requires a case-by-case review, potentially allowing for clarification as to the events leading to report submission and the subject of the reports. This is perhaps best done at a local level given the international, and potential intranational, variation in adverse event reporting practices and the levels and subsequent monitoring of pharmaceuticals in the environment (Ågerstrand et al. 2015; Wang et al. 2019; Jose et al. 2020; Khan et al. 2020; Wilkinson et al. 2022; Gildemeister et al. 2023). This approach would further limit the number of cases of suspected adverse events to assess, but it is probably a necessary initial step. The observed spikes in annual reports for certain PTs could provide an interesting next step of investigation. However, from this global overview of the data it is not possible to examine if these short-term increases are due to a localised environmental event, changes in reporting practices, by chance, or any other possible cause. One possibility is that these increases, and equally any increases in reports for specific medicinal products, may represent a period of stimulated reporting (Hoffman et al. 2014), particularly with a relatively small number of reports. However, under-reporting is more common and is a well understood limitation of pharmacovigilance databases and may be particularly relevant given the acknowledged difficulties in identifying and reporting environment-related adverse events (Gauffin et al. 2023; Holm et al., 2013).

It is important to note that the different levels of reporting for each ATC code or geographical region should not be interpreted as being evidence of increased or decreased risks with these medicine groups, nor that there are good or bad environmental practices in certain areas. This can be highlighted by the relatively low level of reporting of antibiotics (ATC code J01- Antibacterials for systemic use) with 59 reports only. This is despite the high level of attention to the risks of antibiotics in the environment (Bengtsson-Palme et al. 2018; Booton et al. 2021), including the first-ever WHO guidance towards preventing antibiotic pollution during manufacturing directly before the 2024 UN General Assembly discussion on antimicrobial resistance (WHO 2024). However, a thorough review of global legislation and practices regarding ecopharmacology would be beneficial to help provide context.

As discussed earlier there are inherent difficulties in identifying and reporting environment-related adverse events and some level of lateral thinking may be required to improve this. For example, occupation-based environmental exposures were part of the identified HLTs that could potentially have PTs related to environmental exposure, but they were ultimately excluded from the search as they were outside the and may warrant a closer review in the future. Occupational exposure to medicinal products would typically be considered separately to environmental exposures, but there are examples of occupation-related adverse events after unintended exposure or exposure via different means than conventional therapy (Ghatan et al. 2014; Liu et al. 2023) where the type of exposure bears some similarities to environmental exposures. However, occupational environmental exposure is non-equivalent to environmental exposure per se and any hypothetical link between the two is unlikely to represent a straight-line relationship given the complicated nature of exposure in the wider environment.

While this study highlights an important overview of environment-related reporting in VigiBase, that may be of benefit to future research in the area, there are limitations that require consideration. Firstly, with pharmacovigilance data there is always a difficulty in establishing a causal relationship (Uppsala Monitoring Centre, 2025). As these reports were not assessed at a case-by-case basis it was not possible to validate that the search is correctly identifying cases related to environmental exposure. Furthermore, due to a lack of case-by-case analysis it is also not clear what the report is referring to in all cases, for example, how the human was exposed to a medicine within the environment, or even if the report itself was regarding the direct impact on the environment, even if this is less likely given the scope of the database. VigiBase is a living database, and therefore that this overview may not be truly representative for future assessments.

## Conclusion

This study provides the first overview of environment-related reporting of adverse events in an international pharmacovigilance database. The study provides valuable information on the number and content of these reports which will allow greater interpretation and understanding for future research in the area. This is particularly important as the field is still in its relative infancy, and no study has previously explored the potential use of international pharmacovigilance databases as a source of information for the field of ecopharmacovigilance. The total number of reports is growing but remains small and there is conceivable benefit of standardising an approach to collect and collate reports of suspected adverse events related to environmental exposure. However, how to utilise pharmacovigilance to add value to the detection and impact of pharmaceuticals in the environment requires further in-depth assessment. A collaborative effort to share expertise and data from different sectors is likely required to better understand the effects of pharmaceuticals on the environment and any potential effects of humans and animals that interact with the affected environments.

## Supplementary Information

Below is the link to the electronic supplementary material.

(ESM 1) (DOCX 21.8 KB)

## Data Availability

The data that support the findings of this study are not publicly available. Access to the data is restricted based on the conditions for access within the WHO Programme for International Drug Monitoring. Subject to these conditions, some of the data is available from the authors on reasonable request.
