# Supplementary material for: Ecopharmacovigilance and pharmacovigilance: an analysis of environment-related reporting in VigiBase
Source: Environ Sci Pollut Res Int. 2026 Apr 15;33(14):6816–26. doi: 10.1007/s11356-026-37744-6 (PMC13124797; doi:10.1007/s11356-026-37744-6)
Supplement: Supplementary file 1 — (DOCX 21.8 KB) [file 11356_2026_37744_MOESM1_ESM.docx]

**Supplementary Material**

Supplementary Table 1. Counts of reported medicines and vaccines by Anatomical Therapeutic Chemical Classification First and Second Levels for Environmental-related Reporting in VigiBase as of as of July 1st, 2024.

| **ATC First Level** | **ATC Second Level** | **Count (n=1,968)** | **Percentage** |
| --- | --- | --- | --- |
| **A** |  | **145** | **7.4** |
|  | A01 | 27 | 1.4 |
|  | A02 | 15 | 0.8 |
|  | A03 | 5 | 0.3 |
|  | A04 | 14 | 0.7 |
|  | A05 | 3 | 0.2 |
|  | A06 | 5 | 0.3 |
|  | A07 | 23 | 1.2 |
|  | A08 | 1 | 0.1 |
|  | A10 | 34 | 1.7 |
|  | A12 | 10 | 0.5 |
|  | A15 | 2 | 0.1 |
|  | A16 | 6 | 0.3 |
| **B** |  | **53** | **2.7** |
|  | B01 | 19 | 1.0 |
|  | B02 | 3 | 0.2 |
|  | B03 | 2 | 0.1 |
|  | B05 | 29 | 1.5 |
| **C** |  | **132** | **6.7** |
|  | C01 | 24 | 1.2 |
|  | C02 | 20 | 1.0 |
|  | C05 | 72 | 3.7 |
|  | C07 | 6 | 0.3 |
|  | C08 | 4 | 0.2 |
|  | C09 | 2 | 0.1 |
|  | C10 | 4 | 0.2 |
| **D** |  | **302** | **15.3** |
|  | D01 | 9 | 0.5 |
|  | D02 | 46 | 2.3 |
|  | D03 | 7 | 0.4 |
|  | D04 | 52 | 2.6 |
|  | D05 | 1 | 0.1 |
|  | D06 | 46 | 2.3 |
|  | D07 | 14 | 0.7 |
|  | D08 | 13 | 0.7 |
|  | D09 | 6 | 0.3 |
|  | D10 | 13 | 0.7 |
|  | D11 | 95 | 4.8 |
| **G** |  | **83** | **4.2** |
|  | G01 | 26 | 1.3 |
|  | G02 | 25 | 1.3 |
|  | G03 | 12 | 0.6 |
|  | G04 | 20 | 1.0 |
| **H** |  | **25** | **1.3** |
|  | H01 | 3 | 0.2 |
|  | H02 | 10 | 0.5 |
|  | H03 | 10 | 0.5 |
|  | H05 | 2 | 0.1 |
| **J** |  | **237** | **12.0** |
|  | J01 | 59 | 3.0 |
|  | J02 | 4 | 0.2 |
|  | J05 | 6 | 0.3 |
|  | J06 | 9 | 0.5 |
|  | J07 | 159 | 8.1 |
| **L** |  | **116** | **5.9** |
|  | L01 | 35 | 1.8 |
|  | L02 | 6 | 0.3 |
|  | L03 | 8 | 0.4 |
|  | L04 | 67 | 3.4 |
| **M** |  | **47** | **2.4** |
|  | M01 | 14 | 0.7 |
|  | M02 | 18 | 0.9 |
|  | M03 | 9 | 0.5 |
|  | M05 | 5 | 0.3 |
|  | M09 | 1 | 0.1 |
| **N** |  | **298** | **15.1** |
|  | N01 | 16 | 0.8 |
|  | N02 | 85 | 4.3 |
|  | N03 | 25 | 1.3 |
|  | N04 | 10 | 0.5 |
|  | N05 | 72 | 3.7 |
|  | N06 | 56 | 2.8 |
|  | N07 | 34 | 1.7 |
| **P** |  | **18** | **0.9** |
|  | P01 | 13 | 0.7 |
|  | P03 | 5 | 0.3 |
| **R** |  | **175** | **8.9** |
|  | R01 | 41 | 2.1 |
|  | R02 | 12 | 0.6 |
|  | R03 | 80 | 4.1 |
|  | R05 | 15 | 0.8 |
|  | R06 | 19 | 1.0 |
|  | R07 | 8 | 0.4 |
| **S** |  | **177** | **9.0** |
|  | S01 | 91 | 4.6 |
|  | S02 | 49 | 2.5 |
|  | S03 | 37 | 1.9 |
| **V** |  | **104** | **5.3** |
|  | V01 | 1 | 0.1 |
|  | V03 | 26 | 1.3 |
|  | V04 | 30 | 1.5 |
|  | V07 | 24 | 1.2 |
|  | V08 | 6 | 0.3 |
|  | V09 | 1 | 0.1 |
|  | V10 | 1 | 0.1 |
|  | V90 | 8 | 0.4 |
|  | V91 | 7 | 0.4 |
| - |  | 56 | 2.8 |
